# 

**Published:** 1881-08

**Authors:** 


### NATIONAL DENTAL ASSOCIATION.

The Dental Association of the United States of America will hold its regular annual meeting in Republican Hall, 55 West Thirty-third street, near junction Broadway and Sixth avenue, New York City, commencing on Monday, August 8th, 1881, at eleven o'clock, A. M.

At the Sturtevant House, a very fine hotel, near to the place of meeting, persons attending this meeting will be entertained at reduced rates, viz., on the European plan, $1 per day ; or the American plan, $2.50 per day. The "Coleman House," opposite, also has rooms at $1 per day. Board or rooms can be secured by addressing either the hotel people, White's Dental Depot, or Frank M. Odell, Chairman of the Executive Committee, West 38th street.

Officers.—*President.*—A. L. Northrop, New York, N. Y.; *First*

*Vice-President.*—Frank Abbott, New York, N. Y.; *Second Vice-President.*—F. A. Levy, Orange, N. J.; *Third Vice-President.*—J. B. Patrick, Charleston, S. C.; *Secretary.*—R. Finley Hunt, Washington, D. C.; *Assistant Secretary.*—J. H. Smith, New Haven, Conn.; *Treasurer.*—H. B. Noble, Washington, D. C.; *Assistant Treasurer.*—F. M. Odell, New York, N. Y.

Committee on Correspondence with U. S. Government.—R. B. Winder, Baltimore, Md.; W. H. Dwindle, New York, N. Y.; John Allen, New York, N. Y.; V. E. Turner, Raleigh, N. C.; J. Curtiss Smithe, Washington, D. C.

Executive Committee.—F. M. Odell. New York, N. Y.; John Allen, New York, N. Y.; J. C. Sproull, New York, N. Y.

### ARTICLE III.

### MEMBERSHIP.

SECTION I. Any dentist may be elected to membership of this Association upon application, accompanied by credentials of membership in a State society, or by a recommendation from five members of this association, or of his State society.

A simple statement of the objects of this association is set forth in the Constitution.

One object was to have a broadly National body, both theoretically and practically, and thus "give to the profession in every part of the country a proper share in its control and in its benefits." Another was, "by concentrating the strength of the profession in one truly National body," and by uniting in its membership the best talent of that profession in the whole country, to induce the United States Government to extend to this organization all the aid it properly could, in the collection of facts and statistics, so necessary to the establishment of the truths of any science.

These were and are the main objects, as set forth in the Constitution adopted, and in the report accompanying it when submitted; and there are no selfish or sinister ones.

You are cordially invited to unite with us in carrying out this great work. Come, see for yourself, and aid, both at home and in our meetings, in furthering all good objects and in defeating all unworthy ones.

R. FINLEY HUNT, D. D. S.,

*Secretary.*

### HYMENEAL.

" Domestic happiness, thou olny bliss
Of Paradise that has survived the fall."

WORKMAN—BULLOCK.—The marriage of Dr. William Hunter Workman, to Miss Fanny Bullock, daughter of ex-Gov. Bullock, took place at All Saint's Church, Worcester, Mass., on 16th June, 1881, in the presence of a brilliant assemblage of friends.

SCHWARTZE—BURKART.—On June 15, by the Rev. John D. Foley, D. D., at St. Martin's Church, Dr. John Schwartze, to Miss Sue R. Burkart, both of Baltimore. No cards.

VAN BIBBER—LUSBY.—On June 22, at the rectory of St. Ann's Church, New York, by Monsignore T. S. Preston, Dr. John Van Bibber to Mary F. Lusby, both of Baltimore.

SHIPLEY—YOUNG.—On June 23, by the Rev. J. A. Regester,

Dr. D. McG. Shipley, of Baltimore County, to Fannie G. Young, daughter of the late James Young, of Baltimore. No cards.

RAMBO—JONES.—Samuel D. Rambo, D. D. S., of Rio, Brazil, to Miss Emma H. Jones, of Ga., U. S., on the 12th of May, 1881, at Bahia.

We congratulate our good friend and former student on this nuptial event, and wish that the band of love which now unites the joyous pair may be one of endless happiness.

WILL *YOU* PLEASE SEND IN *YOUR* SUBSCRIPTION MONEY *NOW?*

*We take the privilege of deciding what we shall publish, but the author only is responsible for his production.*

We have facilities which enable us to make liberal discounts to our cash subscribers on text and many other books, also Webster's Unabridged Dictionary, the various publications of Frank Leslie and Harper, Scribner's Monthly, Lippincott's Magazine, North American Review, Popular Science Monthly, &c., &c., also many of the leading daily and weekly news and pictorial papers.

AN EXTRA
INDUCEMENT
*To Subscribers.*
See the Advertisement of the Johnson Revolving Book Case in the May issue.

This column will be sold for advertising space six months during the year at *special rates.*

Money is at OUR RISK ONLY when sent by Post Office Money Order, Registered Letter, or Check on Banks in New York City, made payable to INDEPENDENT PRACTITIONER, 20 Courtlandt street, N. Y. City.

If you prefer to send check on your private bank, unless located in the city [illegible]

